# Detecting Contamination or Trends in the Concentrations of Trace Metals in Marine Environments

**DOI:** 10.6028/jres.093.061

**Published:** 1988-06-01

**Authors:** Eric A. Crecelius

**Affiliations:** Battelle/Marine Research Laboratory, 439 West Sequim Bay Road, Sequim, WA 98382

## 1. Introduction

Three marine monitoring programs now in progress were designed to detect contaminated marine ecosystems and to quantify temporal trends in contaminant concentrations. These programs are the National Status and Trends “Mussel Watch” Program, which is funded by NOAA’s Ocean Assessment Division, and the Beaufort Sea Monitoring Program and California Outer Continental Shelf Phase II Monitoring Program, which are both funded by the U.S. Department of Interior Minerals Management Service. As part of these monitoring programs, replicate samples of sediments and organisms from numerous stations are collected annually and analyzed for contaminants.

## 2. Field Sampling Methods

### 2.1 Sampling Rationale

Critical to the success of monitoring programs is the minimization of sampling variability. The sampling strategy was developed to effectively achieve the following:

- ensure uniformity of sampling techniques through establishment of and adherence to specific detailed field protocols;
- collect organisms from indigenous populations in areas considered integrative of contaminant inputs;
- collect undisturbed, depositional surface sediments from areas considered integrative of contaminant inputs;
- employ collection methods that minimize contamination;
- employ sample position-fixing techniques accurate to ± 100 m or better;
- sample for auxiliary parameters (shell length, sediment grain size, organic carbon) that may be used to normalize the variability of analytical data;
- plan subsequent collections during the same season and from precisely the same site coordinates.

### 2.2 Sediment Sampling Methods

Sediment was collected at three or more replicate stations within a site using a Kynar-coated grab sampler or box corer designed to collect undisturbed surficial sediment. Prior to subsampling the surface sediment, the quality and integrity of the sample was determined according to specified criteria. A grab sample was acceptable if it contained overlying water (siphoned prior to subsampling), and was not acceptable if the sampler over-penetrated the sediment. A Kynar-coated stainless steel sediment scoop was specifically designed to collect uncontaminated, undisturbed sediment from the grab sampler.

### 2.3 Organism Sampling Methods

In sampling for bivalves, the primary objective was to obtain three discrete samples from three stations within each site, representing site replicates. However, at sites where this was not possible, a pool of bivalves representative of the site also constituted an acceptable sample. Pooled site samples or composites were generated when bivalves were collected subtidally or when the distribution of intertidal populations did not permit discrete samples to be collected. Due to the low abundances of bivalves in some areas, alternate species, such as amphipods, crabs, and gastropods, were collected in baited epoxy-coated commercial steel minnow traps.

## 3. Analytical Methods

### 3.1 Analytical Rationale

The success of monitoring programs depends equally on both the design and execution of the analytical effort. The goal of the analytical program is to provide data of the highest quality via state-of-the-art techniques, provide documentation of the quality attainable (accuracy and precision), and improve the sensitivity of trend assessment by minimizing analytical variability. Based on these goals, the analytical strategy included the following objectives:

- ensure interlaboratory comparability of analytical techniques and provide method validation through participation in intercalibration exercises;
- ensure interlaboratory analytical uniformity through specific establishment of and adherence to detailed analytical protocols;
- document the quality of the data generated (based on observed limits of detection, precision, and accuracy) through adherence to detailed quality assurance protocols;
- measure auxiliary parameters such as shell length in bivalves, grain size, and total organic carbon in sediments in an effort to normalize contaminant data, correcting for variability resulting from biological, physicochemical, and geochemical processes.

### 3.2 Sediment Analysis

Sediment samples were freeze-dried and blended in a Spex mixer-mill, then 4 g were ground in a Spex ceramic ball mill. A 0.5-g aliquot of ground sediment was pressed into a 2-cm-diameter pellet and analyzed by energy dispersive x-ray fluorescence (XRF) [1] for Al, As, Ba, Cr, Cu, Fe, Mn, Ni, Fb, Si, V, and Zn. For the metals analyzed by atomic absorption, 0.2-g aliquots of the dry homogenate were digested with 4:1 nitric acid/perchloric acid in Teflon digestion bombs in an oven for 4 hours. After these samples were allowed to cool, hydrofluoric acid was added and the digestion bombs were returned to the 130 °C oven for 8 to 12 hours. The next day, boric acid was added to the solutions and the bombs were returned to the 130 °C oven for 8 hours. After cooling, solution volumes were calculated and the solutions stored in polyethylene vials until analyzed. Mercury was analyzed by cold vapor atomic absorption similar to the method of Bloom and Crecelius [2]. The other metals (Ag, Cd, Sb, Se, Sn and Tl) were analyzed by Zeeman graphite furnace.

### 3.3 Tissue Analysis

All samples were sized and shucked prior to tissue processing. Tissue samples were freeze-dried to a constant weight and ground to a powder in a plastic mixer-mill. Half-gram aliquots of dry tissue homogenate from each station sample were reserved for XRF analysis for As, Cu, Fe, Mn, Se, Si, and Zn. For all other analyses, half-gram aliquots of dry tissue homogenate from each station sample were weighed in an acid-cleaned, preweighed Teflon digestion bomb. These samples were predigested at 50 °C for 4 hours without the bomb sealed, using 4:1 nitric acid/perchloric acid, then sealed and digested at 130 °C for 4 hours. After cooling, samples were diluted with deionized-distilled water. Solution volumes were calculated and the sample solutions transferred to polyethylene vials for analysis. The analytical methods used to determine specific metals included cold vapor atomic absorption for Hg, hydride for Sn, and Zeeman graphite furnace for Ag, Al, Ba, Cd, Cr, Ni, Pb, Sb, and Tl.

## 4. Results and Discussion

Determining the intrasite variability for specific chemical parameters is a key step in the process of evaluating the incremental change that one can expect to detect at a given site. The determined variability includes both the intrasite or sampling variability and the analytical variability.

The intrasite variability for trace metals in sediment usually has a coefficient of variation (CV) from 5% to 20%. However, the variability occasionally exceeds 50%. The variability appears to be primarily related to the intrasite variability in grain size, and secondarily related to analytical detection limits for some elements.

Measurements of mud content (silt plus clay) at several sites had a CV of greater than 100%. Usually, trace metal CVs at these sites were also very high, in the range of 40% to 80%. This relationship between the high CVs for mud and trace metals is not surprising because of the positive correlation of these metals with fine grain sediments.

The concentrations of the crustal elements (Al, Fe, Mn, and Si) are less influenced by grain-size variations than are the trace elements and, therefore, had the lowest intrasite CVs, usually less than the 10%.

The intrasite CV has a direct effect on the ability to detect either temporal trends or between-site differences. Statistical calculations (table 1) indicate that with a site replicate sample number of 3 and an intrasite CV of 0.1, a 1.4-fold difference in concentration would be detectable with 80% confidence using a two-sided t-test at the 0.05 significance level.

Intrasite variability for metals in bivalves is similar to that for sediments. Commonly, CV values for metals range from 10% to 20%, indicating that relatively small incremental changes in metal concentrations can be detected. For example, a 1.4-fold or 40% change can be detected if four site replicate samples have a CV of 0.1 (table 1).

The temporal trends in Ba concentrations in California shelf sediments have been examined. Barium was analyzed at 13 stations both in October 1986 and January 1987. A paired t-test was used to test if the 13 station Ba means were different in October than in January. The means (724 and 738 *μ*g/g Ba) were not significantly different at an *α*=0.05. Assuming the variance in these data is a good estimate for future differences between the 13 stations, then an absolute difference of about 25 *μ*g/g Ba, or a 4% change in Ba, could be detected with an *α*=0.01. The power of the test used was *p* =0.95, or the probability that we reject the null hypothesis when we should.

## Figures and Tables

**Table 1:** Minimum number of replicates necessary to detect a K-fold difference in geometric means with 80% confidence, using a two-sided t-test at significance level 0.05. Coefficient of variation of measurement (equals analytical plus sampling variability)

K^a^	0.1	0.2	0.3	0.4	0.5
1.1	19	71	157	278	433
1.2	6	20	44	77	120
1.3	4	11	22	38	58
1.4	3	7	14	24	36
1.5	3	5	10	17	25
1.6	3	5	8	13	19
1.7	3	4	7	10	15
1.8	2	4	6	9	13
1.9	2	3	5	8	11
2.0	2	3	5	7	10

a 1.4=40% change in value; 2.0=100% change (i.e., twofold change in value, etc.).
